# Subcutaneous emphysema following a minor dental procedure: a case report

**DOI:** 10.1186/s13256-026-06164-y

**Published:** 2026-06-01

**Authors:** Mohammad Sadegh Rajabian, Reyhaneh Naseri, Hadiseh Mahram, Parviz Mardani

**Affiliations:** 1 Department of Surgery, Shiraz University of Medical Sciences, Shiraz, Iran; 2 Thoracic and Vascular Surgery Research Center, Shiraz University of Medical Science, Central Building of Shiraz University of Medical Sciences, Zand St., Shiraz, Iran; 3 School of Medicine, Shiraz University of Medical Sciences, Shiraz, Iran

**Keywords:** Complications, Dental restoration, Iatrogenic disease, Restorative treatment, Subcutaneous emphysema

## Abstract

**Background:**

Subcutaneous emphysema (SE) is a rare but well-documented complication following various dental procedures. Diagnosis and early detection of SE is important because it may lead to serious and potentially life-threatening complications and infections.

**Case presentation:**

This case report presents a 32-year-old Iranian female with right-sided cervicofacial pain and swelling accompanied by chest tightness and a sensation resembling “bubble wrap under the skin” after restorative filling of the lower right second molar without using an air turbine drill. The patient was diagnosed with SE following a dental filling.

**Conclusion:**

This report highlights that SE can occur even without high-speed instrumentation, underscoring the need for caution after any dental procedure that compromises the mucosa. Given the increasing worldwide incidence and serious risks of SE, we detail its clinical presentation, treatment approaches, and follow-up protocols. Our aim is to add to the evidence base for managing this rare but serious entity.

## Background

Subcutaneous emphysema (SE) is an infrequent yet serious condition that is frequently underestimated and associated with head and neck trauma, general anesthesia, infections, and various dental and oral surgical procedures [1–3]. Typical clinical presentations include facial swelling and pain, which can be mistaken for allergic reactions or odontogenic infections [4]. Accurate, timely diagnosis, incorporating both clinical assessment and radiological imaging, is needed, as SE may lead to life-threatening complications, such as tension pneumothorax, pneumopericardium resulting in cardiac tamponade, air embolism, tracheal compression, and mediastinitis. Rapid differentiation of these conditions is critical in emergency settings [5, 6].

We present a notable case of cervical SE following a routine mandibular right second molar restoration performed without an air turbine drill, emphasizing that the risk of SE extends beyond procedures involving high-speed instrumentation. Our objective is to elucidate the diagnostic pathway, reinforce management principles, and contribute to the existing literature on this rare but consequential entity.

## Case presentation

A 32-year-old Iranian female presented to the emergency department of our tertiary care center with right-sided cervicofacial pain and swelling, accompanied by chest tightness. She also reported feeling small, plastic air pockets, like bubble wrap, under the skin of the right side of the face and neck. She mentioned that she had undergone a routine dental filling of the right second molar a day before her admission. The procedure was performed under local anesthesia with a local Xylocaine® (plus epinephrine) injection, without ventilation or continuous positive airway pressure. The dentist used a low-speed handpiece and performed a direct composite resin restoration. An air turbine drill was not used for the filling.

She had no pre-existing cardiopulmonary conditions and was in good health; her medical history included asthma and minor thalassemia. There was no record of head or neck pathologies, although she had undergone rhinoplasty two years prior. No recent trauma, surgery, infections, or indications of barotrauma were reported. She denied any other medical conditions or use of regular medications.

On examination, vital signs were as follows: blood pressure 120/70 mmHg, heart rate 82 bpm, respiratory rate 19, oxygen saturation 96%, and body temperature 36.5 °C. Intraoral inspection revealed a recently restored lower right second molar without gross decay, infection, or open wound. She had slight right-sided swelling in the periorbital, buccal, and submandibular regions without associated erythema. The affected areas also exhibited crepitus and mild tenderness on palpation. Lung fields were clear on auscultation. The patient had normal phonation, with no evidence of airway obstruction or respiratory distress. The remainder of the physical examination was within normal limits.

**Fig. 1:**
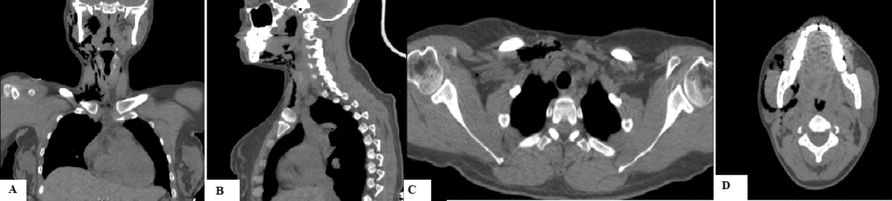
Neck and chest computed tomography shows air accumulation involving: (**A**), coronal view; in the right side supraclavicular fossa, unilateral neck, and buccal regions; (**B**), sagittal view; neck and submandibular region; (**C**), axial view; upper part of chest; (**D**), axial view, right side buccal region

Laboratory findings showed a white cell count of 4300, a hemoglobin level of 8.9 g/dL, a platelet count of 264,000/μL, and a mean corpuscular volume of 64 Fl. Other biochemical parameters were within normal ranges. The initial computed tomography (CT) of the thoracocervicofacial region confirmed SE on the right side of the masticator space, buccal space, parapharyngeal space, sublingual space, parotid space, and prevertebral space. However, there was no evidence of fluid collection (Fig. 1). The patient was diagnosed with SE subsequent to a dental filling performed without an air turbine drill.

Based on the clinical and radiological findings of SE, the possibility of necrotizing fasciitis could not be ruled out. The patient was immediately hospitalized for bed rest and airway monitoring. A conservative management approach was employed, with expectations of natural resorption of the air. Prophylactic empirical intravenous antibiotic therapy consisting of ceftriaxone (1gr every 12 h) and clindamycin (600mg every 8 h) was administered to prevent infection. No surgical intervention was required.

After three days, the patient's clinical condition improved, with a marked reduction in swelling and crepitus on palpation. Subsequently, the patient was discharged with oral antibiotics, including ciprofloxacin (500mg every 12 h) and clindamycin (every 8 h) for a total of four days. In the first month of follow-up, she was doing well, with no sequelae, and had no complaints.

## Discussion and conclusions

SE is defined as air entering subcutaneous areas and tissues, which may spread along fascial planes to the periorbital, mediastinal, pericardial, and/or thoracic spaces. Even some gas-forming organisms may also be an origin of SE [2]. Its consequence may cause any life-threatening physiologic problems such as tension pneumomediastinum, pneumothorax, pneumopericardium, or even air embolism [3, 7, 8]. This report describes the case of a 32-year-old woman presenting with cervicofacial pain and swelling, accompanied by chest tightness, following restorative dental fillings, along with its management. Our aim is to contribute to the existing literature by emphasizing the importance of early recognition by emergency physicians and meticulous patient monitoring to ensure favorable outcomes.

Although iatrogenic SE is a key differential diagnosis for facial and mediastinal swelling, it is often overlooked by physicians due to limited awareness of this complication post-dental procedures. Clinicians should consider SE in patients presenting with facial or neck swelling and pain who have recently undergone dental interventions, as failure to recognize it may lead to life-threatening complications. A thorough history, clinical examination—including palpation for crepitations—and appropriate imaging, such as contrast-enhanced or non-contrast CT, are essential for accurate diagnosis and assessment of SE expansion. It is also important to maintain a broad differential diagnosis, considering less common but serious conditions like necrotizing fasciitis or mediastinitis, which require prompt identification to prevent catastrophic outcomes.

It is now known that SE can be caused by the inappropriate use of high-speed air-driven handpieces. In most dental procedures, the leading procedure is dental removal, especially of 3rd lower molars [9–12]. The recent systematic review by Jones et al. found that 51.1% of SE cases are linked to air-driven handpieces, and a further 9.6% are specifically linked to air syringes used near a mucosal breach. Restorative procedures accounted for 28% of those air-drill cases. Our case clearly falls outside that majority [9]. Similarly, the clinical study by Saito et al. of 11 cases noted that 9 involved a device that emits air (air turbine, handpiece, or air syringe) [13]. Our case is an outlier in these cohorts, a clear example of SE following a low-speed restorative procedure without any air-driven instrument or syringe. This highlights a vital point, confirming that although air pressure is a major risk factor, the fundamental mechanism is simply the introduction of air through a breach, regardless of how it occurs.

Roots of first, second, and third molars communicate directly to sublingual and submandibular spaces, which makes them more susceptible to developing SE because of the anatomical connection to these spaces [2, 14]. Moreover, preparations, filling treatments of mandibular molar regions, and any disruption of the dentoalveolar membrane can also lead to these difficulties [15]. Symptoms may not always occur immediately; SE can develop and progress within minutes to hours after dental procedures and might take more than a day or two [2, 11, 12]. In our case, the involved tooth was also the right second molar, following a routine dental filling.

Treatment of SE is typically conservative, with close monitoring of patients’ vital signs during the hospital stay to enable early detection of life-threatening complications. Although SE is relatively benign, resolving within 2–10 days, and infection is rare, prophylactic broad-spectrum antibiotics with oral flora coverage are frequently prescribed to reduce infection risk [12, 13], [16, 17]. Therefore, in a well patient, SE is usually self-limited, and additional treatment beyond securing the airway and monitoring circulation is not needed.

We should, however, acknowledge the limitations of this report. Primarily, we lack an intraoral photograph of the restoration site and a follow-up CT scan to visually document resolution. An intraoral image would have provided useful correlative detail, although our clinical exam explicitly noted the restoration. A follow-up CT was not medically indicated, given her rapid and complete clinical recovery; we therefore relied on physical examination to confirm resolution, which is standard in most conservative management pathways. These gaps, while not undermining the core clinical narrative, highlight areas for more systematic data collection in future case reports.

Dentists and primary care physicians should be aware of SE and consider it when encountering patients who present with swelling or pain in the face, neck, and chest after any dental procedure, to achieve the correct diagnosis as soon as possible. Moreover, close monitoring of patients after diagnosis is crucial to identify further complications and to manage treatment promptly.

## Data Availability

All data generated or analyzed during this study are included in this published article.

## Abbreviations

CT: Computed tomography
SE: Subcutaneous emphysema
